# Adjustment for physical activity in studies of sedentary behaviour

**DOI:** 10.1186/s12982-015-0032-9

**Published:** 2015-07-09

**Authors:** Andrew Page, Geeske Peeters, Dafna Merom

**Affiliations:** Centre for Health Research, School of Medicine, University of Western Sydney, Campbelltown, Australia; School of Public Health, The University of Queensland, Brisbane, Australia; School of Science and Health, University of Western Sydney, Campbelltown, Australia

**Keywords:** Sedentary behaviour, Physical activity, Methods, Measurement, Collinearity

## Abstract

Sedentary behaviour (too much sitting, as distinct from too little exercise) has emerged as a potentially significant public health issue. Analytically, researchers have reported ‘independent’ associations between sedentary behaviour (SB) and a number of health outcomes by adjusting for physical activity (PA) (and other confounders), and conclude that SB is associated with the outcome even in those who are physically active. However, the logical rationale for why adjustments for PA are required is often not delineated, and as a consequence, PA has been conceptualised as a confounder, an intermediary, and an effect measure modifier—sometimes simultaneously—in studies of SB and health outcomes. This paper discusses the analytical assumptions underlying adjustment for PA in studies of SB and a given outcome, and considers the implications for associations between SB and health.

## Background

In the past decade physical activity and public health research has shifted its focus to the harms of ‘sedentary behaviour’ (SB). Sedentary behaviour (too much sitting, as distinct from too little physical activity) has emerged as a potentially significant public health issue, given its associations with morbidity [1], and all-cause and cause-specific mortality [2–5]. ‘Too much sitting’ has been described as an independent risk factor of chronic disease, irrespective of amounts of moderate to vigorous physical activity (MVPA), possibly due to differential effects on health outcomes. SB and physical activity relate differently to health outcomes and via distinct biological mechanisms, [6, 7] and it is possible to have both high SB and MVPA in a 24-h period.

The protective effect of MVPA on health outcomes relates to improvements in cardio-respiratory fitness through increased oxygen supply to the myocardium and improved myocardial contraction [8], as well as lower blood pressure [9], improved lipoprotein profile [10], and increased insulin sensitivity [11]. The main biological mechanism proposed for SB relates to cardio-metabolic changes associated with decreased lipoprotein lipase activity (associated with increases in plasma triglycerides and decreases in HDL-cholesterol), which are associated with coronary heart disease, type II diabetes and obesity [12]. It is hypothesised that chronic exposure to SB reduces skeletal muscle contractile activity which evokes a process of suppressing the amount of capillary lipoprotein lipase in the muscle [13].

However, the way in which these two exposures are specified in analyses is often confused, and does not necessarily relate to clear biological mechanisms. Physical activity (commonly operationalized as achieving the recommended MVPA for health) has been considered as a confounder, intermediary, and effect measure modifier, sometimes simultaneously [1] in studies of sedentary behaviour and health outcomes. The logical rationale for why adjustment for physical activity is required is often not clearly articulated, nor is there always a clear delineation of the temporal ordering of measurement periods of SB and PA. This paper discusses the analytical assumptions underlying adjustment for PA in studies of SB and a given outcome, and considers the implications for associations between SB and health outcomes.

## Causal assumptions underlie simultaneous adjustment

**Figure 1:**
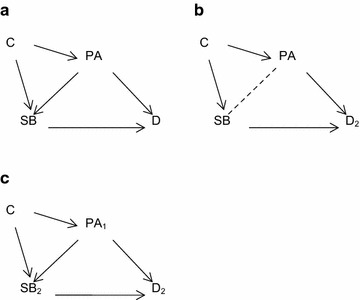
Physical activity as a *common cause* (confounder) of sedentary behaviour and disease outcomes. **a** PA is a common cause of SB and the outcome (*D*).* C* is an exogenous variable that is a common cause of both PA and SB. **b** The causal direction between PA and SB is unknown. *C* is an exogenous variable that is a common cause of both PA and SB. **c** PA is a common cause of SB and the outcome (*D*), with sub-scripts denoting measurement at time 1 and time 2. *C* is an exogenous variable that is a common cause of both PA and SB.

**Figure 2:**
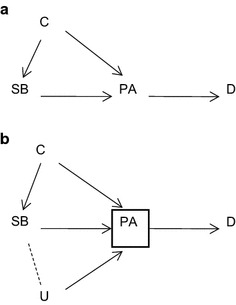
Physical activity as an *intermediary* between sedentary behaviour and disease outcomes. **a** PA is an intermediary between SB and D. **b** Adjustment for PA as an intermediary induces an association between SB and unmeasured confounders (*U*) by conditioning on the common effect PA.

Analytically, researchers obtain ‘independent’ associations between sedentary behaviour (SB) and a given outcome by adjusting for physical activity (PA), and conclude that SB is associated with the outcome even in those who are physically active (that is, achieving recommended MVPA). By including PA and SB simultaneously in a statistical model, there is an implied causal assumption, illustrated by the directed acyclic graphs in Figures 1 and 2. If PA is considered a common cause (confounder) of SB and the outcome (D), the implication is that PA causes SB (Figure 1a), and adjustment is necessary. There may also be an exogenous variable (C), such as injury or disability status, which is a common cause of both PA and SB, and where the causal direction between PA and SB is unknown. In this case adjustment for PA is necessary if C is unmeasured and PA is not collinear with SB (Figure 1b).

If PA is considered an intermediary between SB and D, then the implication is that PA is an effect of SB, and adjustment is conducted to demonstrate the extent of mediation, and the ‘independent’ effect of SB on D (Figure 2a). This latter adjustment is problematic—as for all mediation analyses using conditional models—as adjustment for PA as an intermediary induces an association between SB and unmeasured confounders (U) by conditioning on the common effect of PA (Figure 2b) (known as collider stratification bias) [14, 15]. Unless there are explicitly measured temporal relationships between PA and SB (Figure 1c), where people do more (or less) MVPA at time 1 and therefore become more (or less) sedentary at time 2, then conceptualising PA as a confounder or intermediary assumes a causal direction that may not be warranted based on the putative aetiological mechanisms by which PA and SB affect health outcomes.

**Figure 3:**
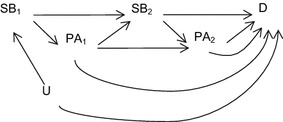
Physical activity as a *confounder* and *intermediary* between sedentary behaviour and disease outcomes. Sub-scripts denote measurement at time 1 and time 2.

Additionally, studies usually do not explicitly delineate the temporal ordering of SB and PA measures, and do not analytically address the inter-dependence of SB and PA across time: SB is a time-dependent exposure that is analysed in the presence of the time-dependent co-variate of PA. In this context, PA can be considered as both a confounder and an intermediary, where PA is an effect of SB but is also a common cause of SB and D (Figure 3). If conceptualising PA as a time-dependent co-variate of SB (a time-dependent exposure) is a more appropriate reflection of the putative aetiological mechanisms by which PA and SB affect health outcomes, then marginal structural models (MSMs) [16] can be used to estimate the ‘independent’ effects of SB on a given health outcome. MSMs provide a solution to the collider stratification bias inherent in conditional approaches to adjustment for PA in studies of SB and a given outcome (Figure 2). MSMs derive inverse probability weights to re-weight a dataset to reflect the probability of a given level of PA based on observed level of SB. This approach allows unbiased estimation of the marginal association between SB and D, adjusting for PA.

## Biological interaction, statistical interaction, or collinearity

**Figure 4:**
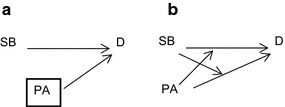
Physical activity as an *effect measure modifier* of associations between sedentary behaviour and disease outcomes. **a** The association between SB and D differs in magnitude within strata of PA. **b** Model of effect measure modification proposed by Weinberg [20].

An alternative conceptualisation, and the reason for why SB might be considered separately to PA, is that SB has a different aetiological pathway to D, and that the presence of some level of PA or SB affects the level of the other. That is, the relationship is one of effect measure modification due to the interaction between plausible biological mechanisms (Figure 4). For example, perhaps reductions in lipoprotein lipase (LPL) activity affect oxidative muscle fibres in response to SB (with subsequent deleterious effects on metabolic health), whereas increases in LPL affect glycolytic fibres in response to PA (with subsequent beneficial effects on metabolic health) [6].

A number of observational studies have considered interactions between PA and SB [5, 17, 18], to show that the magnitude of the association between SB and D differs within levels of PA in associations with disease outcomes. Conceptualising PA as an effect measure modifier of an SB-D association may be a more appropriate causal model, if PA does not cause SB and vice versa. If there are plausible and independent biological mechanisms for PA and SB, then this may be the most appropriate way of conceptualising the separate or synergistic effects of SB and PA on health outcomes. However, if SB and PA are two sides of the same coin (that is, the energy expenditure continuum), then testing effect measure modification here may be analogous to testing effect measure modification by number of cigarette packs smoked per day in the association between smoking status and lung cancer.

Figure 4a shows a causal graph that is compatible with direct effect measure modification [19], and which implies that within strata of PA (for example, sufficient or insufficient MVPA), the association between SB and D differs in magnitude. Some authors have criticised this specification of effect measure modification using causal graphs, as it does not sufficiently capture the myriad ways in which an effect measure modifier may affect an exposure-disease association [20]. Weinberg [20] illustrates the limited way in which causal graphs can specify effect measure modification by proposing a range of alternative models for known biological and environmental interactions, which are not strictly ‘directed acyclic graphs’ in that not all vertices emanating from an ‘ancestor’ terminate at a ‘descendant’ variable, which is a basic condition [21].

However, one model of effect measure modification proposed by Weinberg [20] (Figure 4b), is consistent with the inherent inter-dependence between SB and PA and how SB and PA may independently affect disease outcomes. In this model, the expectation E(D|SB,PA) is not constant in SB for any fixed value of PA, and is not constant in PA for any fixed value of SB. Neither SB nor PA is a cause of the other, however on a given effect measure modification scale for some pair of SB values (SB_1_ and SB_0_) and some pair of PA values (PA_1_ and PA_0_) then there is effect measure modification: E(D|SB_1_,PA_1_) − E(D|SB_0_,PA_0_) ≠ E(D|SB_1_,PA_0_) − E(D|SB_0_,PA_0_)] + E(D|SB_0_,PA_1_) − E(D|SB_0_,PA_0_). Few studies consider statistical interaction between PA and SB on the additive scale [3, 22] which is the most appropriate scale to examine joint effects of plausible biological mechanisms.

It is also important to make the distinction between statistical and biological interaction. Statistical interaction is dependent on an arbitrary choice of scale, whereas biological interaction exists or does not, and can be investigated in joint effects analyses [23]. However, this distinction between statistical and biological interaction is contestable, and is inherently difficult to estimate even with defined biological mechanisms [24, 25]. In instances where PA is specified as an effect measure modifier of SB and D, it is not clear whether this reflects statistical interaction or biological interaction given the often self-reported nature of exposure measurement and the inherent collinearity between SB and PA categorised over a 24 h time period [26]. All dimensions of activity during a 24-h period (i.e. MVPA, light intensity PA, standing, SB, and sleep) may be associated with health outcomes, however, simultaneous adjustment of these dimensions of activity and/or assessment of substitution of effects (of SB to PA or vice versa) is problematic given this collinearity [26].

In studies where self-reported measures of PA and SB are used, this inherent collinearity may not be explicit, given that PA measures are often restricted to MVPA (often in one activity domain) ensuring a notionally non-identifiable relationship between PA and SB (although light intensity PA and total PA can also be included in analyses as residual confounders). If time spent in all intensity levels is measured using, for example, an accelerometer for a given 24-h period, then there is an explicitly identifiable relationship between PA and SB measures.

## Should any adjustment be made?

If PA is not a common cause of SB and D, and is not an effect of SB on the causal pathway to D (an intermediary), then adjustment for PA in models of SB and D is not required, in either standard conditional models or marginal structural models. If there is evidence of statistical interaction between SB and PA then further research should establish whether this interaction is an artefact of the arbitrary selection of statistical scale, or whether this represents biologically distinct causal pathways for SB and PA associated with D. If SB is simply the complementary (and identifiable) component of PA in the total 24 h period of energy expenditure, and not dependent on the presence of the other (and not an effect measure modifier), then it is unclear as to why analyses would need to consider both PA and SB simultaneously at all.
